# Supplementary material for: Modeling Complex Proton Transport Phenomena—Exploring the Limits of Fine-Tuning and Transferability of Foundational Machine-Learned Force Fields
Source: J Phys Chem C Nanomater Interfaces. 2025 May 14;129(21):9662–9. doi: 10.1021/acs.jpcc.5c02064 (PMC12128250; doi:10.1021/acs.jpcc.5c02064)
Supplement: Supplementary file 1 [file jp5c02064_si_001.pdf]

Supplementary Information for "Modelling complex proton transport phenomena - Exploring the limits of fine-tuning and transferability of foundational machine-learned force fields "

Malte Grunert[1,2†], Max Großmann[1,2†], Jonas Hänseroth[3], Aaron Flötotto[3], Jules Oumard[3], Johannes Laurenz Wolf[3], Erich Runge[1,2], Christian Dreßler[3*]

[1]Theoretical Physics I, Institute of Physics, Technische Universität Ilmenau, 98693 Ilmenau, Germany.

[2]Center of Micro- and Nanotechnologies, Technische Universität Ilmenau, 98693 Ilmenau, Germany.

[2]Theoretical Solid State Physics, Institute of Physics, Technische Universität Ilmenau, 98693 Ilmenau, Germany.

*Corresponding author(s). E-mail(s): christian.dressler@tu-ilmenau.de;
†These authors contributed equally to this work.

This supporting information provides details on: (i) Additional figures visualizing the radial distribution functions (RDF) obtained from MD simulation using different MACE models. We also show the similarity coefficients (SC) between the MACE and AIMD simulations for all models. (ii) Error metrics for forces and energies after training for each MACE model used in this study. (iii) Model performance against AIMD simulations. (iv) A figure illustrating the Arrhenius analysis of temperature-dependent diffusion coefficients used to extract the proton-diffusion activation energy. (v) A table reporting the average number of proton jumps per hydrogen atom per picosecond in both AIMD and fine-tuned MACE simulations

Supplementary Note 1:
Similarity coefficients of Radial Distribution functions

As already mentioned in the main text, comparing F and F+D3 in Supplementary Fig. S1, we find that the inclusion of the D3 dispersion correction does not significantly change the RDFs. This is due to the fact that the intermolecular interactions of the present system are dominated by strong hydrogen bonds, for which dispersion forces do not play a major role.

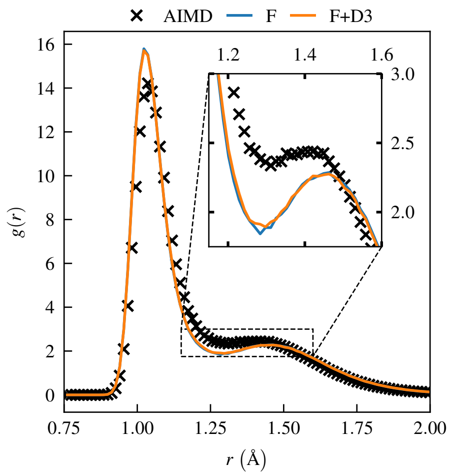

Fig. S1: Extension of Fig. 1b of the main text, comparing the RDF of the MACE foundation model with and without the empirical D3 dispersion correction [1].

Table S1: Systematic comparison of the radial dispersion function $g(r)$ for all bond pair combinations in CDP using the similarity coefficient SC as defined in Eq. (1) in the main text with respect to the AIMD results, i.e., SC[MACE; AIMD]. The last column shows the SC averaged over all bond pair combinations in the row. The top two rows show the results for the small MACE foundation model (F) and the small MACE foundation model with D3 dispersion correction (F+D3). The bottom four rows show the results for MACE models fine-tuned to AIMD trajectories of different time lengths, e.g., FT@1ps (see main text for details).

SC[MACE; AIMD]	Cs-Cs	Cs-H	Cs-O	Cs-P	H-H	H-O	H-P	O-O	O-P	P-P	Average
F	0.934	0.908	0.940	0.864	0.903	0.912	0.899	0.961	0.933	0.965	0.922
F+D3	0.921	0.923	0.945	0.883	0.925	0.917	0.906	0.965	0.937	0.961	0.928
FT@0.01ps	0.919	0.967	0.965	0.933	0.940	0.977	0.972	0.980	0.983	0.976	0.961
FT@0.1ps	0.979	0.974	0.968	0.926	0.967	0.974	0.991	0.981	0.972	0.963	0.969
FT@1ps	0.963	0.966	0.964	0.906	0.951	0.973	0.984	0.980	0.974	0.981	0.964
FT@228ps	0.956	0.966	0.964	0.925	0.949	0.974	0.990	0.980	0.978	0.979	0.966
FT@CPP	0.941	0.970	0.968	0.918	0.953	0.981	0.979	0.980	0.975	0.976	0.964

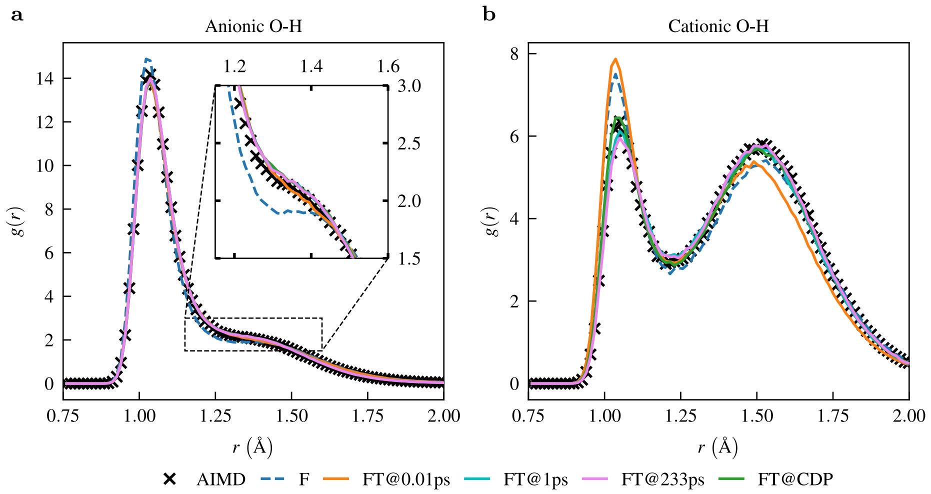

Fig. S2: Extension of Fig. 2b and c of the main text, showing more RDFs obtained from MD simulation using different MACE models for CPP. **a** Comparison of the anionic O-H radial distribution function $g(r)$ obtained from different MACE models (see main text for details) with $g(r)$ from an AIMD simulation. The inset highlights the peak of $g(r)$ at $d_{\mathrm{OH}} = 1.5$ Å, which is commonly referred to as the "short strong hydrogen bond" or "low/barrier hydrogen bond". **b** Comparison of the cationic O-H radial distribution function $g(r)$ obtained from different MACE models (see main text for details) with $g(r)$ from an AIMD simulation. See Fig. 2a in the main text for a visualization of anionic $H_2PO_4^-$ and cationic $H_4PO_4^+$ tetrahedra.

Table S2: Similarity coefficient SC between $g(r)$ from different MACE models and the AIMD simulation for the anionic O-H, cationic O-H bonds, and the average over all bond pair combinations in CPP. Here, the model FT@CDP refers to fine-tuned model FT@228ps from Supplementary Table S1, which was fine-tuned to an AIMD trajectory of CDP (see main text for details).

	F	FT@0.01ps	FT@0.1ps	FT@1ps	FT@233ps	FT@CDP
Anionic O-H	0.947	0.973	0.982	0.986	0.987	0.983
Cationic O-H	0.941	0.926	0.950	0.974	0.976	0.981
Average	0.933	0.911	0.939	0.973	0.974	0.969

Supplementary Note 2: Error metrics for forces and energies after training

Table S3: Table summarizing the final training error metrics for energy (E) and force (F) for different models for CDP. Each row corresponds to a model trained on a different number of snapshots (see main text for details). The final-error metrics on the training data after training include the root mean square error (RMSE) for energies and forces, respectively.

	RMSE E (meV^{-1} atom^{-1})	RMSE F (meV^{-1} Å^{-1})	relative F RMSE (%)
FT@0.01ps	0.8	34.2	3.56
FT@0.1ps	0.2	45.5	4.7
FT@1ps	2.0	32.7	3.42
FT@228ps	1.1	21.9	2.27

Table S4: Table summarizing the final training error metrics for energy (E) and force (F) predictions for different models for CPP. Each row corresponds to a model trained on a different number of snapshots (see main text for details). The final-error metrics on the training data after training include the root mean square error (RMSE) for energies and forces, respectively.

	RMSE E (meV^{-1} atom^{-1})	RMSE F (meV^{-1} Å^{-1})	relative F RMSE (%)
FT@0.01ps	1.2	50.0	4.87
FT@0.1ps	1.7	51.6	5.28
FT@1ps	0.7	27.8	2.84
FT@233ps	0.7	22.7	2.3

Supplementary Note 3: Model performance against DFT simulations

Table S5: Table comparing the predicted energies (E) and forces (F) for CDP from MACE models with explicit DFT calculations over 235 equally spaced frames extracted from an 11 ns trajectory generated by MACE. The selected snapshots are independent of the training data, ensuring an unbiased evaluation. Each row represents a different version of the MACE model (see main text for details). The comparison includes root mean square error (RMSE) metrics for energies and forces, respectively.

	RMSE E (meV^{-1} $atom^{-1}$)	RMSE F (meV^{-1} $Å^{-1}$)	relative F RMSE (%)
Foundation	307525	248	23.5
FT@0.01ps	0.6	132	13.4
FT@0.1ps	0.68	94.2	9.8
FT@1ps	2.34	59.3	6.1
FT@228ps	0.38	37.1	3.8
FT@CPP (= FT@233ps)	1483	40.9	4.2

Table S6: Table comparing the predicted energies (E) and forces (F) for CPP from MACE models with explicit DFT calculations over 800 equally spaced frames extracted from a 4 ns trajectory generated by MACE. The selected snapshots are independent of the training data, ensuring an unbiased evaluation. Each row represents a different version of the MACE model (see main text for details). The comparison includes root mean square error (RMSE) metrics for energies and forces, respectively.

	RMSE E (meV^{-1} $atom^{-1}$)	RMSE F (meV^{-1} $Å^{-1}$)	relative F RMSE (%)
Foundation	292000	232	22
FT@0.01ps	17.6	143	14.5
FT@0.1ps	1.3	94.5	9.8
FT@1ps	0.2	50.8	5.2
FT@233ps	0.2	33.7	3.5
FT@CDP (= FT@228ps)	1172	45.1	47

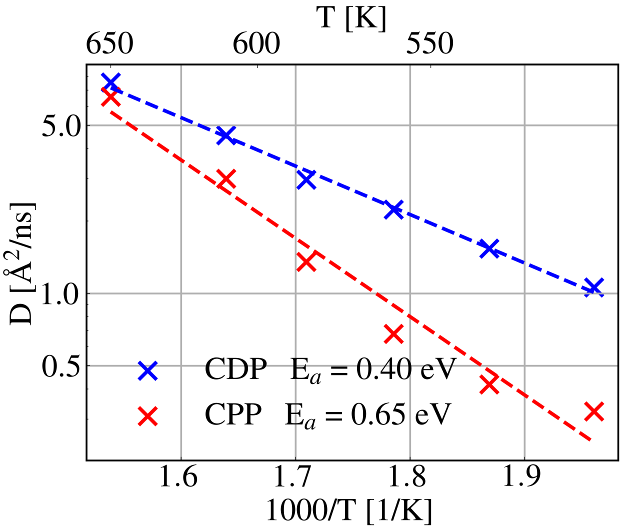

Fig. S3: Determination of the proton diffusion activation energy

Supplementary Note 4: Proton diffusion activation energy calculation

At the end of the 'Results' section, we discuss the trends in the activation energy for proton diffusion. To calculate the activation energy, we assume the following temperature dependence of the diffusion coefficient:

$$D(T) = A \exp(-\frac{E_a}{k_b T})$$

with prefactor A, temperature T, and activation energy E_a. In Figure S3, we plot $ln(D)$ against $\frac{1}{T}$ and obtain the diffusion coefficient from the linear slope $m = -\frac{E_a}{k_b}$

Supplementary Note 5: Average number of proton jumps per hydrogen atom and per ps

Table S7: Average number of proton jumps per hydrogen atom and per ps.

compound	CPP	CDP
AIMD	15.1	13.4
fine-tuned MACE	14.1	14.1

References

1. Grimme, S., Antony, J., Ehrlich, S. & Krieg, H. A Consistent and Accurate Ab Initio Parametrization of Density Functional Dispersion Correction (DFT-D) for the 94 Elements H-Pu. *J. Chem. Phys.* **132,** 154104 (2010).
